# Tandem CAR T-cells targeting CD19 and NKG2DL can overcome CD19 antigen escape in B-ALL

**DOI:** 10.3389/fimmu.2025.1557405

**Published:** 2025-05-09

**Authors:** Jennifer Bolsée, Benjamin Violle, Céline Jacques-Hespel, Thuy Nguyen, Caroline Lonez, Eytan Breman

**Affiliations:** R&D Department, Celyad Oncology, Mont-Saint-Guibert, Belgium

**Keywords:** multispecific CAR, tandem CAR, B-ALL, antigen escape, antigen heterogeneity, CD19, NKG2DL

## Abstract

**Introduction:**

Chimeric antigen receptor (CAR) T-cell therapies have achieved remarkable success in treating B-cell malignancies, including acute lymphoblastic leukemia (B-ALL). However, despite high remission rates, relapse due to antigen escape remains a significant challenge. To overcome this, designing CAR T-cells targeting multiple cancer antigens simultaneously is a promising strategy. NKG2D ligands (NKG2DL) are eight stress-induced ligands expressed by cancer cells but largely absent on healthy cells.

**Methods and Results:**

We hypothesized that simultaneous targeting of NKG2DL (using the NKG2D extracellular domain) and CD19 can prevent CD19 antigen escape and improve long-term remission rates in B-ALL patients. We developed three tandem CARs targeting both CD19 and NKG2DL and demonstrated that two tandem candidates were highly effective against both CD19+ and CD19- cancer cell lines. Importantly, when compared to CD19 CAR T-cells, tandem CAR T-cells exhibited comparable cytokine secretion, cytolytic activity and proliferation levels when incubated with cancer cells expressing CD19 and were still effective when incubated with cancer cells lacking CD19. Moreover, T-cells transduced with the selected CD19/NKG2DL tandem CAR were functional against CD19+ primary B-ALL samples and controlled tumor growth in a highly challenging xenograft model representing a CD19- B-ALL relapse.

**Discussion:**

These findings provide proof-of-concept that NKG2D-based tandem CARs offer a promising approach to overcome antigen escape and enhance anti-tumor efficacy in B-cell malignancies.

## Introduction

CD19-targeting chimeric antigen receptor (CAR) T-cells are now approved for treating specific relapsed/refractory (r/r) B-cell malignancies such as B-cell acute lymphoblastic leukemia (B-ALL), B-cell non-Hodgkin lymphoma (B-NHL) and B-cell chronic lymphocytic leukemia (B-CLL) (1–5). However, despite impressive clinical activity, some patients are refractory, and others eventually relapse. Mechanisms of relapse in B-cell malignancies, including B-ALL, have been extensively studied, with CD19 escape identified as a major cause, along with the lack of CAR T-cell persistence (6–8). During oncogenic transformation, genetic instability creates tumor variants with different adaptation levels to their environment (9). Thus, the immune pressure exerted by a monospecific or single CAR-T therapy can lead to the emergence of antigen-negative or antigen-low tumor cells. This can occur through two main mechanisms: 1) lineage switch, where patients relapse with a genetically related but phenotypically different malignancy (10, 11), and 2) acquired mutations or splice variants leading to CD19 isoforms lacking the target epitope or to the absence of CD19 on the cell surface (12). Patients relapsing with a CD19-negative disease have very poor prognosis, hence there is an urgent need to develop novel CAR T-cell therapies targeting alternative antigens or targeting multiple antigens simultaneously.

Recognition of multiple antigens can be achieved through various strategies. The first consists in co-infusing or infusing sequentially several monospecific CAR T-cell products. The second consists in the infusion of T-cells transduced with two viral vectors, giving rise to a mix of single and multispecific CAR T-cells. Alternatively, T-cells can be transduced with a single viral vector encoding tandem or dual (bicistronic) receptors. In the tandem design, two antigen-binding moieties (often in the form of scFv’s) are connected via a linker and incorporated into a single CAR protein. In the dual approach, two independent monospecific CAR proteins are co-expressed on the surface of T-cells.

Preliminary clinical results with CD19 multispecific CARs highlight areas for improvement in the design of next-generation tandem CAR T-cells. First, the ideal second antigen should be highly expressed. Next, the selected engager should bind this antigen with sufficient affinity. Finally, tandem designs should minimize steric hindrance between the two engagers and ensure accessibility of each engager to its target epitope (13–15).

NKG2D ligands (NKG2DL) represent an attractive family of targets for multispecific CAR approaches. This family is composed of eight different stress-induced ligands (MICA, MICB and ULBP1-6) that are induced upon cellular damage, cell transformation or viral infection but are largely absent from the surface of healthy cells (16, 17). NKG2DL recognition by the NKG2D receptor expressed by NK cells and some subsets of T-cells allows for the surveillance against oncogenic transformation and viral infection (18–20). NKG2DL were shown to be expressed in hematological indications such as acute myeloid leukemia (AML) (21, 22), B-cell leukemias and lymphomas (20, 23–26). In addition, the susceptibility of primary B-ALL cells to NK cell activity was also shown to be dependent on the NKG2D-NKG2DL axis (20, 27), suggesting that CD19/NKG2DL multispecific CAR T-cells might represent an interesting approach to prevent CD19 antigen escape in B-cell malignancies.

Although autologous CAR T-cell therapies proved to be effective in B-cell malignancies, the time, cost, and manufacturing failure risk associated are significant disadvantages. Off-the-shelf allogeneic approaches offer a solution to these challenges and should increase the accessibility of CAR T-cell treatments to a larger population cohort.

Here, we demonstrate that allogeneic CAR T-cells expressing tandem receptors targeting CD19 and NKG2DL show equivalent efficacy to CD19 single-targeting CAR T-cells against CD19+ cancer cell lines and primary B-ALL samples. Importantly, this antitumor activity was fully conserved in the absence of the CD19 antigen. Overall, we provide the proof-of-concept that NKG2D can be combined with a CD19-targeting scFv in the context of a tandem receptor to overcome antigen escape, as an alternative to currently evaluated tandem receptors.

## Material and methods

### Study design

The objective of this study was to evaluate the functionality of different CD19/NKG2DL tandem candidates against CD19+ and CD19- cancer cell lines and CD19+ primary B-ALL samples *in vitro* and to demonstrate their anti-tumor efficacy in CD19- B-ALL relapse model *in vivo*. All *in vitro* experiments, otherwise mentioned, were performed with CAR T-cells produced from PBMCs isolated from five healthy donors. For the *in vivo* study, a total five NSG mice (except in the control group, n=3) were randomized into treatment groups before T-cell injection based on body weight and tumor load. With this model, previous experiments have shown that this number of mice is sufficient to ensure reproducibility and to highlight significant differences.

### Cell lines and primary samples

Nalm-6 cells (human B-cell precursor leukemia) were purchased from DSMZ. HeLa (human cervix adenocarcinoma), Phoenix ECO (human embryonic kidney) and PG13 cells (mouse embryonic fibroblast) were purchased from American Type Culture Collection (ATCC). Nalm-6 were cultured in RPMI-1640 (21875091, ThermoFisher) supplemented with 10% heat-inactivated fetal bovine serum (10438026, Life technologies), 1% GlutaMAX™ (3505006, ThermoFisher), 1% penicillin/streptomycin (15140148, ThermoFisher). HeLa were cultured in DMEM (DMEM-HA, Westburg) supplemented with 10% heat-inactivated fetal bovine serum (10438026, ThermoFisher), 1% GlutaMAX™ (3505006, ThermoFisher), 1% penicillin/streptomycin (15140148, ThermoFisher). Nalm-6 Luciferase/GFP (Luc/GFP) were generated by transduction of Nalm-6 with a lentiviral vector coding for a GFP tag, a firefly luciferase and a puromycin resistance (System Biosciences). HeLa NucLight Red were generated by transduction with a lentivirus coding for a nuclear-restricted Red Fluorescent protein (NucLight Red) and a puromycin resistance (4625, Sartorius). Nalm-6 CD19 KO cells were generated by nucleofection of the Alt-R^®^ Cas9 enzyme, the Alt-R^®^ Cas9 electroporation enhancer and the crRNA:tracrRNA duplex targeting human CD19 locus (target sequence: 5’-CGGGCCACAGCTCAAGACGC-3’) (Integrated DNA Technologies) with the 4D Nucleofector™ System (Lonza). HeLa NucLight Red CD19 were generated by transduction of HeLa NucLight Red with a γ-retrovirus coding for a truncated form of CD19 together with an hygromycin resistance gene. Cells were maintained in a humidified atmosphere containing 5% CO_2_ at 37°C. Deidentified primary human B-ALL bone marrow specimens were obtained from the CHU UCL Namur site Godinne. Additional information regarding the primary samples is provided in the table below.

### Plasmid construction

Retroviral vectors were generated using the pSFG backbone. CD19 CAR was generated using the CD19 specific FMC63 scFv fused to hinge (55 aa) and transmembrane domains from CD8α. NKG2DL CAR was generated using the ectodomain of NKG2D (aa 82-216) fused to a short hinge (12 aa) and transmembrane domain of CD8α. In CD19/NKG2DL tandem CAR constructs, FMC63 scFv and NKG2D ectodomain were linked via 3, 4 or 5 repetitions of a glycine-serine linker (G_4_S)_3_, itself fused to the transmembrane domain of CD8α via a short hinge (12 aa) derived from IgG4, a short hinge (12 aa) derived from CD8α or a long hinge (55aa) from CD8α. All receptors carry the intracellular domain of 4-1BB and CD3ζ as co-stimulatory and stimulatory domains and a truncated CD34 (tCD34) as selection marker. All CAR-encoding constructs also contained a shRNA duplex targeting MICA/B and CD3ζ transcripts.

### Retroviral vector production

Retroviral vectors were generated in two steps. Phoenix ECO packaging cells were transiently transfected with the retroviral plasmid of interest and the retroviral vector obtained was used to infect the PG13 packaging cells. Stable PG13 producer cells were seeded, and final retroviral vector was collected 48 and 72 hours post seeding.

### CAR T-cell production

PBMCs from healthy donors were activated with TransAct (130-111-160, Miltenyi) on day 0 and transduced on retronectin (T100B, Takara Bio Europe)-coated plates with a retroviral vector in presence of Akt inhibitor (5773/50, R&D Systems). On day 6, transduced T-cells were enriched with a CD34 selection (130-046-702, Miltenyi) and then expanded until day 10 in presence of AKTi. Finally, TCRα/β positive cells were depleted (130-133-896, Miltenyi) and the final product was cryopreserved. Cultures were performed in X-VIVO™15 (02-60Q, Lonza) supplemented with 5% Human Male AB serum (515-HI, Access cell culture), 1% GlutaMax (3505006, ThermoFisher) and IL-2 (170-076-147, Miltenyi).

### Flow cytometry

Flow cytometry experiments were performed using antibodies directed against human CD4 (560158, BD Biosciences), CD8 (561453, BD Biosciences), CD19 (562441, BD Biosciences), CD25 (53-0259-42, ThermoFisher), CD34 (343516, Biolegend), CD45RA (564552, BD Biosciences), CD62L (25-0629-42, ThermoFisher), CD69 (555533, BD Biosciences), CD107a (328620, Biolegend), CD279 (12-2799-42, ThermoFisher), TIGIT (372714, Biolegend) and TCRα/β (130-113-527, Miltenyi). NKG2DL were detected using antibodies directed against human MICA (FAB1300P, R&D systems), MICB (FAB1599P, R&D systems), ULBP1 (FAB1380P, R&D systems), ULBP3 (FAB1517P, R&D systems), ULBP2-5-6 (FAB1298P, R&D systems) or using a recombinant human NKG2D (rhNKG2D-Fc, 1299-NK-050, R&D systems) detected with an anti-human IgG-Fc antibody (12-4998-82, ThermoFisher). CD19-binding domain was detected using a recombinant human CD19 tagged with a human Fc (rhCD19-Fc, 9269-CD-050, R&D systems) that was recognized by an anti-human IgG-Fc antibody (12-4998-82, ThermoFisher). NKG2DL-binding domain was detected using the CD314 monoclonal antibody (NKG2D (CD314) (568106, BD Biosciences)). CD19- and MICA-binding capacity were evaluated by incubating T-cells with recombinant human CD19 tagged with a human Fc (rhCD19-Fc, 9269-CD-050, R&D systems) recognized by an anti-human IgG-Fc antibody (12-4998-82, ThermoFisher) and with a recombinant human Biotinylated Human MICA Protein, His,Avitag™ (rhMICA-Avi, MIA-H82E6, ACROBiosystems) detected with an Alexa Fluor^®^647 Streptavidin (Biolegend, 405237), respectively. Samples were acquired with an Attune flow cytometer (ThermoFisher) and analyzed with the FlowJo v10.9.0 software.

### Cytokine measurements

Cytokines were measured with ELISA kits from R&D systems (IFN-γ: SIF50 and DY285B; TNF-α: DTA00D; IL-2: S2050) in supernatant from T-cells cultured for 24 hours with cancer cells at 1:1 E:T ratio or with coated ligands. MICA coating was performed by incubating recombinant human MICA (rhMICA-Fc, 1300-MA-050, R&D systems) overnight at 4°C while CD19 coating was performed as described elsewhere (15).

### Cytolytic activity

Cytolytic activity was measured with the Incucyte^®^ S3 system (IC50042, Sartorius) by following the number of Nalm-6 Luc/GFP cancer cells over time. Results are expressed as percentage of remaining cancer cells normalized to t0h timepoint.

### Repeated antigen stimulation assay

T-cells were recursively activated by a co-culture with adherent HeLa NucLight Red or HeLa NucLight Red overexpressing CD19. Every 3 or 4 days, T-cells were harvested and transferred to culture wells seeded with fresh tumor cells, adjusting for a constant viable 1:1 E:T ratio. Upon each round of stimulation, cytotoxic activity was monitored by the Incucyte^®^ S3 system and T cell expansion was assessed by manual counting. Results are expressed as percentage of remaining cancer cells normalized to t0h timepoint.

### Proliferation

For the proliferation assay, T-cells were stained with Cell Trace Violet (CTV) (C34557, ThermoFisher), according to a protocol described elsewhere (28) on day 0, and then cultured in absence or presence of cancer cells at a 1:1 ratio for 4 days. CTV fluorescence was then measured by flow cytometry on live CD4/CD8 gated T-cells.

### Animal model

Mouse experiments were performed in accordance with French and European Regulations and the National Research Council Guide for the Care and Use of Laboratory Animals. Animals were euthanized upon showing symptoms of clinically overt disease (not feeding, lack of activity, abnormal grooming behavior, hunched back posture) or weight loss exceeding 15% compared to the reference day. In this study, healthy female NSG (NOD.Cg-Prkdcscid Il2rgtm1Wjl/SzJ) mice (obtained from Charles River) were i.v. injected on day 0 with 0.1x10^6^ cells of a 1:1 mix of Nalm-6 Luc/GFP: Nalm-6 CD19 KO Luc/GFP. On day 7, mice were i.v. injected with 5x10^6^ CAR T-cells and then i.v. rechallenged with 0.1x10^6^ of Nalm-6 CD19 KO Luc/GFP on day 14, 21 and 28. Tumor load was monitored by bioluminescence imaging (PhotonIMAGER RT, Biospace Lab) and images analyzed using M3Vision image analysis software (Biospace Lab).

### Statistical analysis

Unless otherwise mentioned, data are presented as means + SD or means ± SD. The number of biological replicates, here described as different PBMC donors, is indicated in the figure legend. Statistical analysis was performed on GraphPad Prism 10 software. Datasets from *in vitro* experiments were analyzed by paired, one-way ANOVA, and P values were adjusted for multiple comparisons. Dataset from *in vivo* experiment was analyzed using log-rank Mantel-Cox tests and P values were adjusted for multiple comparisons. Differences with an adjusted P value < 0.05 were considered statistically significant.

## Results

### CD19/NKG2DL tandem receptors efficiently bind their target antigens and do not impact T-cell phenotype

**Figure 1:**
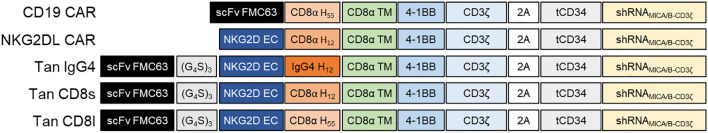
CD19/NKG2DL tandem CAR design. Schematic representation of single and tandem CAR constructs. scFv FMC63, single-chain variable fragment anti-CD19; NKG2D EC, extracellular domain of human NKG2D; (G_4_S)_3_, Glycine-Serine linker; CD8α H_55_, 55 amino acids hinge from human CD8α; CD8α H_12_, 12 amino acids hinge from human CD8α; IgG4 H_12_, 12 amino acids hinge from human IgG4; CD8α TM, transmembrane region from human CD8α; 4-1BB, intracellular domain from 4-1BB; CD3ζ, intracellular domain from human CD3ζ; 2A, 2A self-cleaving peptide; tCD34, truncated CD34; shRNA_MICA/B-CD3ζ_, shRNA duplex targeting MICA/B and CD3ζ.

To assess which allogeneic CD19/NKG2DL tandem CARs would be the most efficient in preventing CD19 antigen escape, we engineered different tandem CARs targeting CD19 and NKG2DL where the CD19 targeting scFv was placed in distal position towards the membrane and linked to the extracellular domain of NKG2D (NKG2D EC) through a glycine-serine linker (G_4_S)_3_ (**Figure 1**). NKG2D EC was bound to a CD8α transmembrane domain (CD8α TM) via a short hinge (12 amino acids) derived from either IgG4 or CD8α (Tan IgG4, Tan CD8s) or via a long hinge (55 amino acids) derived from CD8α (Tan CD8l). The CD19 single-targeting (CD19 CAR) contained the 55 amino acids CD8α hinge (which is similar to the hinge used in Kymriah (tisagenlecleucel) (29)). The NKG2DL single-targeting CAR contained the 12 amino acids CD8α hinge (NKG2DL CAR) to mimic the natural configuration where the extracellular C-type lectin domain of NKG2D is linked to the transmembrane α helix via a short stalk (30, 31). Each CAR construct carried a 4-1BB co-stimulation domain and a CD3ζ activation domain and was co-expressed with a truncated CD34 (tCD34) downstream of a 2A peptide to allow for purification of transduced cells during manufacturing. In addition, the transgene also contained a miRNA-based shRNA duplex targeting MICA/B and the 3’untranslated region (3’UTR) of CD3ζ. We have previously shown that downregulation of CD3ζ eliminates TCRα/β at the surface of T-cells and prevents the risk of GvHD associated to allogeneic cell therapies (32). We have also shown that the inclusion of MICA/B shRNA both prolongs cellular persistence of NKG2DL CAR T-cells and can avoid potential fratricide by avoiding MICA and MICB upregulation upon stress conditions on the surface of CAR T-cells (33).

Activated T-cells were either transduced with a retroviral vector encoding tCD34 tag only (mock), a single-targeting CAR (CD19 or NKG2DL CAR) or a CD19/NKG2DL tandem CAR (using a manufacturing process as summarized in **Supplementary Figure S1A**). Final fold-expansion (**Supplementary Figure S1B**) and percentage of transduced cells were similar for all CAR constructs (between 27.3 and 38.5%, data not shown).

**Figure 2:**
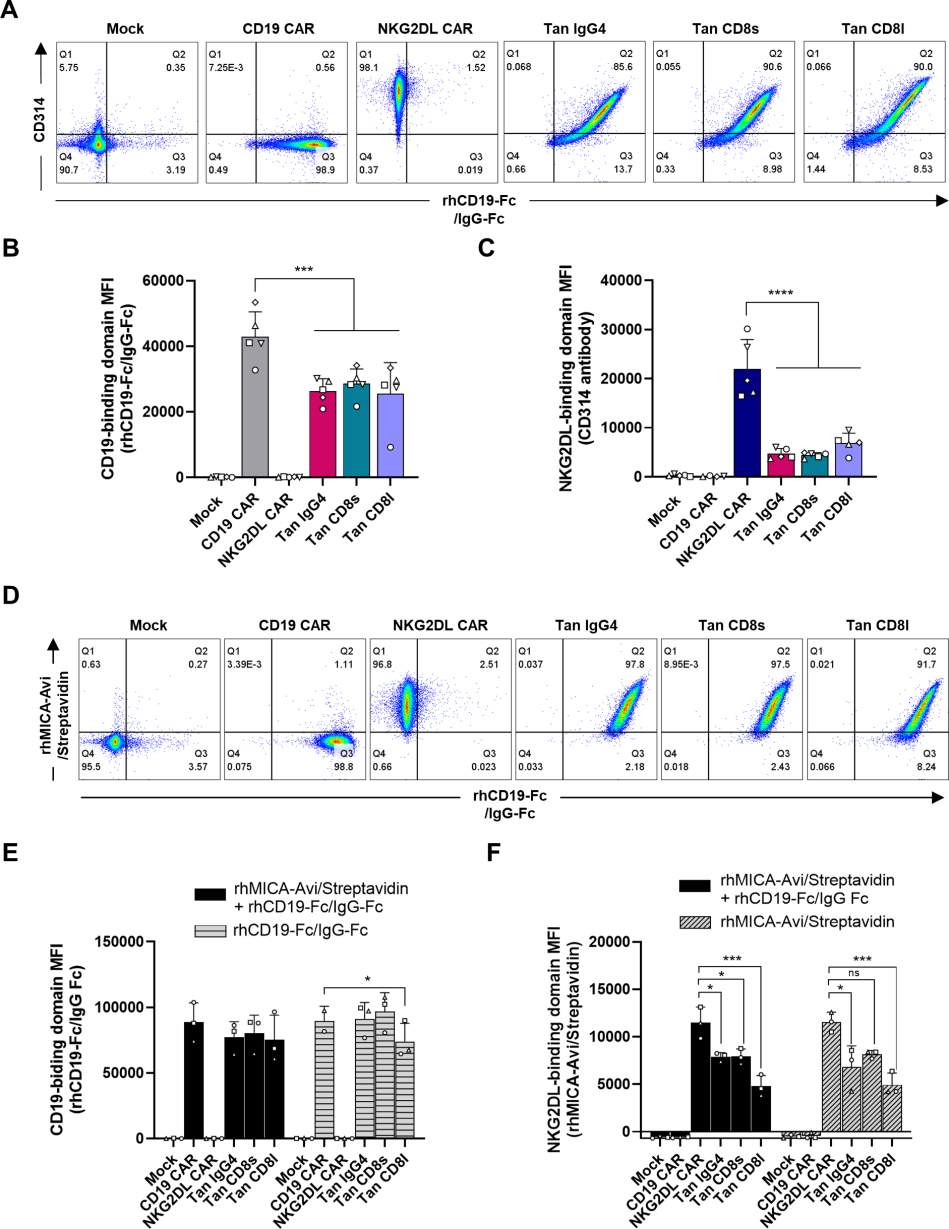
CD19/NKG2DL tandem CAR expression. **(A)** Representative dot plots of CD19- and NKG2DL-binding domain expression detected by staining T-cells with rhCD19-Fc + anti-human IgG-Fc and CD314 antibody. **(B)** CD19-binding domain MFI and **(C)** NKG2DL-binding domain MFI. **(D)** Representative dot plots of CD19- and NKG2DL-binding domain expression detected by staining T-cells with rhCD19-Fc + anti-human IgG-Fc and rhMICA-Avi + streptavidin, respectively, in a subpopulation expressing CD34 marker at similar level. **(E)** CD19-binding domain MFI and **(F)** NKG2DL-binding domain MFI when staining was performed with the two ligands simultaneously (black bars) or with one ligand only (grey dashed bars). Adjusted P values (*P<0.05; ***P < 0.001; ****P < 0.0001) were determined by one-way ANOVA with Dunnet’s correction for multiple comparisons. Data presented as means (SD) of n=5 **(B, D)** or n=3 **(E, F)**. Each symbol denotes a different PBMC donor.

To compare expression of single and tandem CARs, T-cells were incubated with a monoclonal antibody against NKG2D (CD314) and an Fc-tagged CD19 recombinant protein (rhCD19-Fc), which was detected with an anti-human IgG-Fc antibody. Transduced T-cells showed high expression of both CD19- and NKG2DL-binding domains, regardless of the construct (**Figure 2A**). However, the relative expression of the CD19-binding domain was significantly lower in tandem CAR T-cells compared to CD19 CAR T-cells (**Figure 2B**). This reduced expression was linked to lower transgene expression in the tandem CAR T-cells, as indicated by decreased CD34 MFI (**Supplementary Figure S1C**). Similarly, NKG2D expression was lower in tandem CAR T-cells than in NKG2DL CAR T-cells (**Figure 2C**). Only a slight increase in NKG2D MFI was observed when NKG2D staining was performed in absence of rhCD19-Fc (**Supplementary Figures S1D, E**), suggesting that binding of recombinant CD19 to the FMC63 scFv does not substantially reduce accessibility for the NKG2D (CD314) antibody.

To determine whether CD19 binding affects NKG2DL binding and *vice versa*, T-cells were incubated with recombinant MICA (rhMICA-Avi) and/or CD19 (rhCD19-Fc) proteins. As shown in **Figure 2D**, tandem CAR T-cells with comparable transgene expression (i.e. population expressing similar CD34 levels) were able to bind both ligands simultaneously. Furthermore, the binding of one ligand did not impact the binding of the other, as MFIs measured with one or with the two ligands were not statistically different (P=0.4576 for CD19-binding domain and P=0.7461 for NKG2DL-binding domain) (**Figures 2E, F**). However, rhMICA-Avi levels were lower in tandem CAR T-cells compared to NKG2DL single CAR T-cells, even in the absence of rhCD19-Fc. This suggests that the FMC63 scFv hinders access to the NKG2D EC domain.

To reduce this steric hindrance, we increased the linker length to 4 or 5 repetitions of the G_4_S motif between the NKG2D EC and the FMC63 scFv (**Supplementary Figure S2A**). To control for differences in transduction efficiency, CD19- and NKG2DL-binding levels were analyzed in subpopulations with similar CD34 MFIs. However, increasing the linker size had no significant effect on CD19- and NKG2DL-binding levels (**Supplementary Figures S2B, C**). Interestingly, when the positions of FMC63 scFv and NKG2D EC were switched – placing FMC63 scFv proximal and NKG2D EC distal towards the membrane (Tan NKG2D-FMC63.CD8l) – NKG2D detection was restored (**Supplementary Figure S2C**). However, this alteration reduced FMC63 scFv accessibility, as shown by lower levels compared to CD19 single CAR T-cells (**Supplementary Figure S2B**), making this design unsuitable for further development.

Analysis of differentiation and exhaustion markers at harvest showed that both single and tandem CAR T-cells were primarily central memory (CD45RA^-^/CD62L^+^) and effector memory (CD45RA^-^/CD62L^-^) (**Supplementary Figure S3A**), with most of the cells lacking exhaustion markers (**Supplementary Figures S3B, C**). No differences were observed in CD4/CD8 ratio (**Supplementary Figure S3D**). Finally, CD3ζ-targeting shRNA efficiency was confirmed by TCRα/β downregulation in single and tandem CAR T-cells compared to mock-transduced T-cells (**Supplementary Figures S3E, F**). Overall, these data demonstrate that CD19/NKG2DL tandem receptors are highly expressed in primary T-cells, do not alter T-cell phenotype, and bind their respective targets.

### CD19/NKG2DL short hinge tandem CAR T-cells exhibits potent *in vitro* functional activity against both CD19+ and CD19- cancer cells

**Figure 3:**
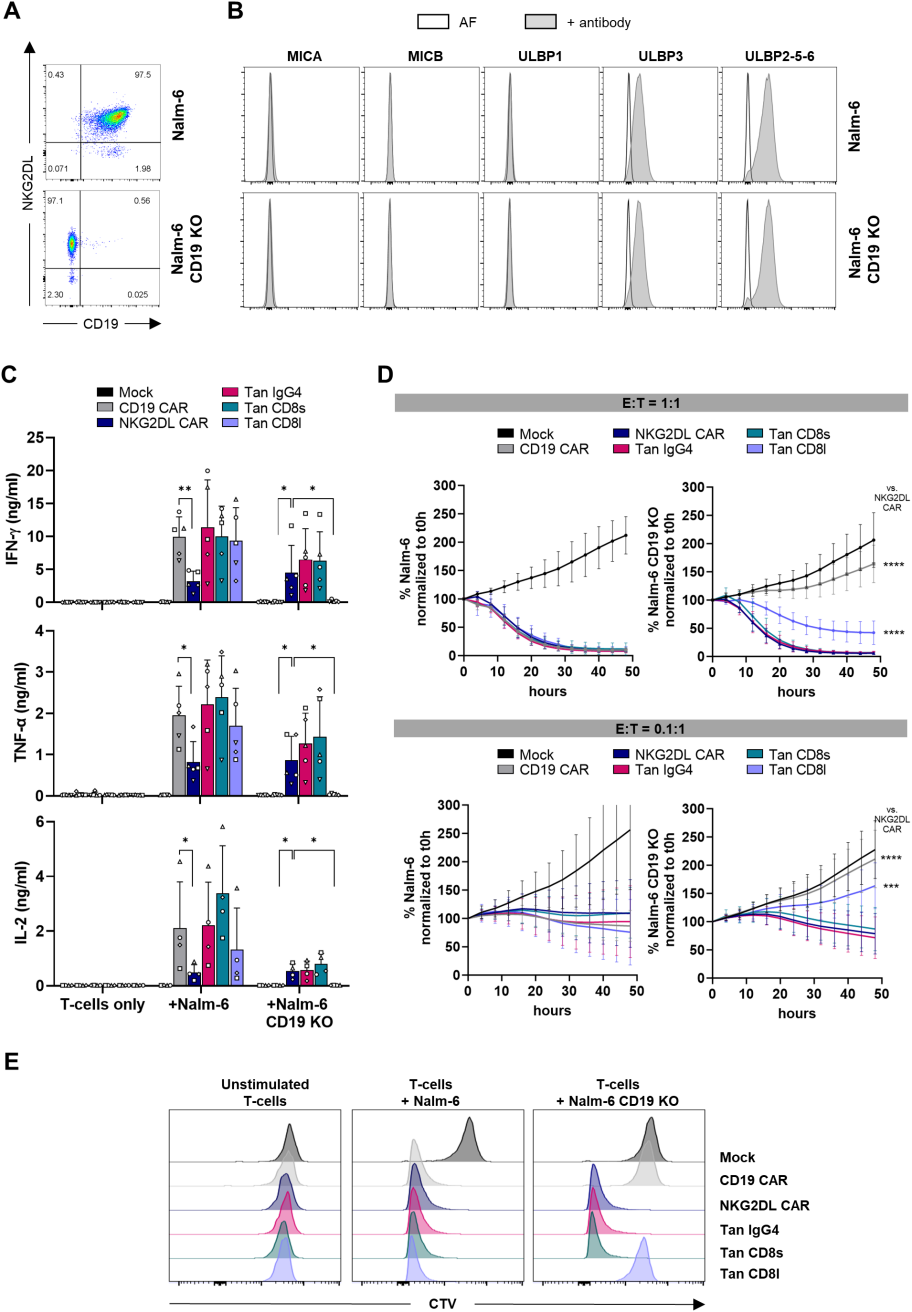
Cytokine secretion, cytolytic activity and proliferation of CD19/NKG2DL tandem CAR T-cells co-cultured with CD19+ and CD19- Nalm-6 cells. **(A)** Expression of CD19 and NKG2DL in Nalm-6 and Nalm-6 CD19 KO cells. **(B)** Expression of individual NKG2DL using antibodies directed against MICA, MICB, ULBP1, ULBP3 or ULBP2-5-6 (grey histograms) *vs* autofluorescence (white histograms). **(C)** Secretion of IFN-γ, TNF-α and IL-2 cytokines after a 24-hour co-culture at 1:1 E:T ratio with Nalm-6 and Nalm-6 CD19 KO cells. **(D)** Cytolytic activity of CAR T-cells against Nalm-6 and Nalm-6 CD19 KO cells at 1:1 and 0.1:1 E:T ratio. Results are expressed as percentage of remaining cancer cells normalized to t0h timepoint. **(E)** Representative experiment showing CTV histograms of CAR T-cells after 4 days of co-culture without cancer cells or at 1:1 E:T ratio with Nalm-6 and Nalm-6 CD19 KO cells (4 individual experiments were performed on 4 different donors). Adjusted P values (*P < 0.05; **P < 0.01; ***P < 0.001; ****P < 0.0001) were determined by one-way ANOVA with Dunnet’s correction for multiple comparisons. Data presented as means (SD) of n=5. Each symbol denotes a different PBMC donor.

To assess the functionality and specificity of CD19/NKG2DL tandem CAR T-cells in the presence and absence of CD19 antigen, we knocked-out the CD19 gene in the B-ALL cell line Nalm-6 (**Figure 3A**). Wild-type and CD19 KO cells expressed ULBP3 and ULBP2-5–6 ligands at similar levels (**Figure 3B**), showing that absence of CD19 does not influence expression of NKG2DL in this cell line.

To evaluate CAR T-cell activity upon antigen recognition, CAR T-cells were co-cultured with Nalm-6 cells and cytokine secretion was measured. All tandem CAR T-cell candidates secreted IFN-γ, TNF-α and IL-2 at similar levels than CD19 single CAR T-cells, indicating that NKG2D EC domain does not interfere with T-cell activation induced by CD19 antigen recognition (**Figure 3C**). As expected, when challenged with CD19 KO cells, cytokine secretion by CD19 single CAR T-cells was abolished. On the other hand, CD19/NKG2DL tandem CAR T-cells carrying a IgG4 or a CD8 short hinge, but not tandem with a CD8 long hinge, conserved their capacity to secrete cytokines in absence of the CD19 antigen, demonstrating that functionality of NKG2D EC domain in a tandem context requires a short hinge. Interestingly, NKG2DL single CAR T-cells secreted significantly lower amounts of cytokines than CD19 and CD19/NKG2DL CAR T-cells when incubated with Nalm-6 cells. It is known that cytokine secretion by CAR T-cells is greatly influenced by antigen density. Hence this difference might be explained by a preference of tandem CAR T-cells to engage the highly expressed CD19 ligand through their high affinity scFv, while NKG2DL CAR T-cells bind ULBP ligands that are probably less expressed with a lower affinity. Although not significant, tandem CAR T-cells incubated with Nalm-6 KO cells tend to secrete lower amounts of IFN-γ while, as expected, NKG2DL CAR secrete similar amounts against both CD19+ and CD19- cell lines.

We next evaluated the cytolytic activity of single and tandem CAR T-cells against Nalm-6 and Nalm-6 CD19 KO cells at different effector to target (E:T) ratios. When challenged at a 1:1 ratio with Nalm-6 cells, both single and tandem CAR T-cells rapidly eliminated cancer cells (**Figure 3D**). In absence of CD19, only NKG2DL single CAR T-cells and T-cells expressing a tandem with a short hinge efficiently eliminated tumor cells, while the tandem with the long hinge showed a significantly lower cytolytic activity. Similarly, at 0.1:1 E:T ratio, incubation of either single or tandem CAR T-cells with Nalm-6 cells led, in all instances, to tumor growth control. Likewise, NKG2DL single and CD19/NKG2DL tandem CAR T-cells with a short hinge lysed CD19 KO cancer cells at this low E:T ratio, while tandem CAR T-cells with the long hinge failed to demonstrate cytolytic activity when co-cultured with CD19 KO cells.

Finally, proliferative capacity of mock, single and tandem CAR T-cells after exposure to Nalm-6 and Nalm-6 CD19 KO cells was evaluated using the Cell Trace Violet (CTV) dye. As shown in **Figure 3E**, all T-cells except mock T-cells proliferated in presence of Nalm-6 cells. When the experiment was performed with Nalm-6 lacking CD19 expression, only T-cells expressing the NKG2DL CAR or a tandem CAR with a short hinge proliferated.

### CD19/NKG2DL tandem CAR T-cells are highly active when stimulated with only CD19 or MICA

**Figure 4:**
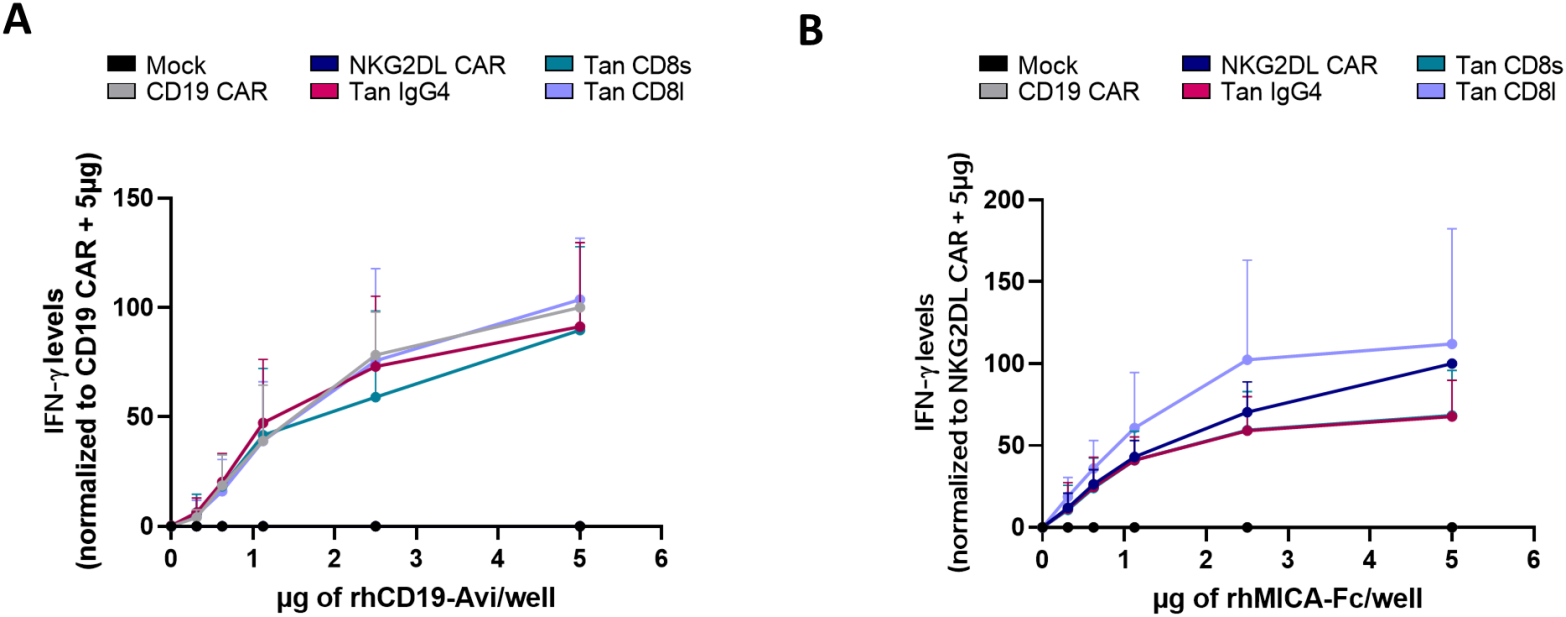
Cytokine secretion of CD19/NKG2DL tandem CAR T-cells upon stimulation with coated CD19 or coated MICA. IFN-γ secretion after a 24-hour exposure with increasing concentrations of **(A)** coated rhCD19-Avi or **(B)** coated rhMICA-Fc.

Previous experiments showed that tandem CAR T-cells with a short hinge region were reactive against cancer cells expressing either CD19 and NKG2DL or NKG2DL alone. However, their functionality against CD19 alone remained untested. Since eliminating NKG2DL would require knocking out 8 genes simultaneously in cancer cells, we instead assessed whether CD19 could activate tandem CAR T-cells using plate-coated CD19. As expected, both CD19 single CAR T-cells and CD19/NKG2DL tandem CAR T-cells secreted IFN-γ in a dose-dependent manner (**Figure 4A**). We also tested whether MICA could activate tandem CAR T-cells using plate-coated MICA. Again, both NKG2DL CAR and tandem CAR T-cells secreted IFN-γ in dose-dependent manner (**Figure 4B**).

These data, together with the previous results, show that CD19/NKG2DL CAR T-cells are reactive against both CD19 and NKG2DL antigens separately.

### CD19/NKG2DL tandem CAR T-cells containing a short hinge maintain high potency in response to chronic antigen exposure

**Figure 5:**
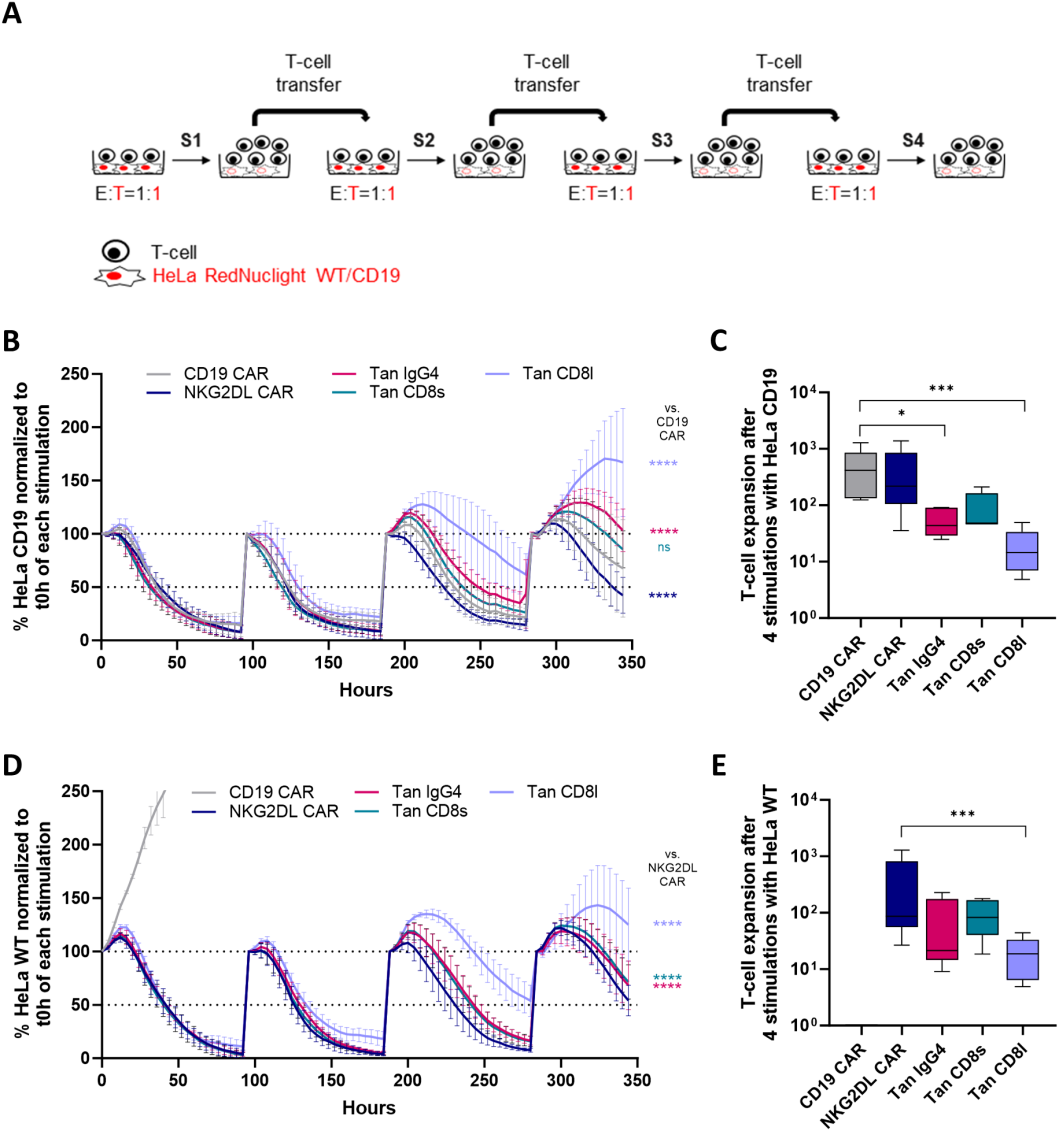
Cytolytic activity and proliferation of CD19/NKG2DL tandem CAR T-cells upon repeated antigen stimulation. **(A)** Experimental design of the repeated antigen stimulation assay **(B)** Cytolytic activity of single and tandem CAR T-cells against HeLa CD19 cells at 1:1 E:T ratio during successive cycles of stimulation. Results are expressed as percentage of remaining cancer cells normalized to t0h timepoint. **(C)** Cumulative expansion of T-cells after four cycles of stimulation with HeLa CD19 cells. **(D)** Cytolytic activity of single and tandem CAR T-cells against HeLa WT cells at 1:1 E:T ratio during successive cycles of stimulation. Results are expressed as percentage of remaining cancer cells normalized to t0h timepoint. **(E)** Cumulative expansion of T-cells after four cycles of stimulation with HeLa WT cells. Adjusted P values (*P < 0.05; ***P < 0.001; ****P < 0.0001) were determined by one-way ANOVA with Dunnet’s correction for multiple comparisons. Data from **(B)** and **(D)** presented as means (SD) of n=5. Data from **(C)** and **(E)** were log-transformed to achieve a normal distribution and presented as box plots with 10^th^ percentile, median, and 90^th^ percentile (n=5). Each symbol denotes a different PBMC donor.

To assess the efficacy of CD19/NKG2DL CAR T-cells under stress conditions, we evaluated their cytolytic activity after chronic antigen exposure (**Figure 5A**). To do so, CAR T-cells were serially co-cultured with HeLa cells either overexpressing CD19 (HeLa CD19) or wild-type (HeLa WT) (**Supplementary Figure S4A**). Several NKG2DL were highly expressed on HeLa WT and HeLa CD19 cells (**Supplementary Figure S4B**).

When HeLa cells expressed CD19, all CAR T-cells rapidly lysed the cancer cells during the first two stimulations (**Figure 5B**). However, from the third stimulation onwards, tandem CAR T-cells with a long hinge were less effective at controlling cancer cell growth compared to other tandem CAR T-cell candidates. This reduced cytolytic activity was accompanied by a lower cumulative expansion compared to CD19 CAR T-cells (**Figure 5C**).

As expected, CD19 single CAR T-cells failed to control tumor cell growth when co-cultured with HeLa WT cells (**Figure 5D**), whereas both tandem CAR T-cells and NKG2DL CAR T-cells maintained tumor control. Similar to observations with HeLa CD19 cells, tandem CAR T-cells with a short hinge exhibited a higher cytolytic activity than tandem CAR T-cells with the long hinge, which correlated with greater proliferative capacity (**Figure 5E**).

Given the widespread use of the CD8α hinge in clinical trials and its slightly superior performance in this experiment, we selected the tandem CAR candidate with the CD8 short hinge for further *in vitro* and *in vivo* evaluation.

### CD19/NKG2DL CD8s tandem CAR T-cells effectively controls antigen escape *in vivo*

**Figure 6:**
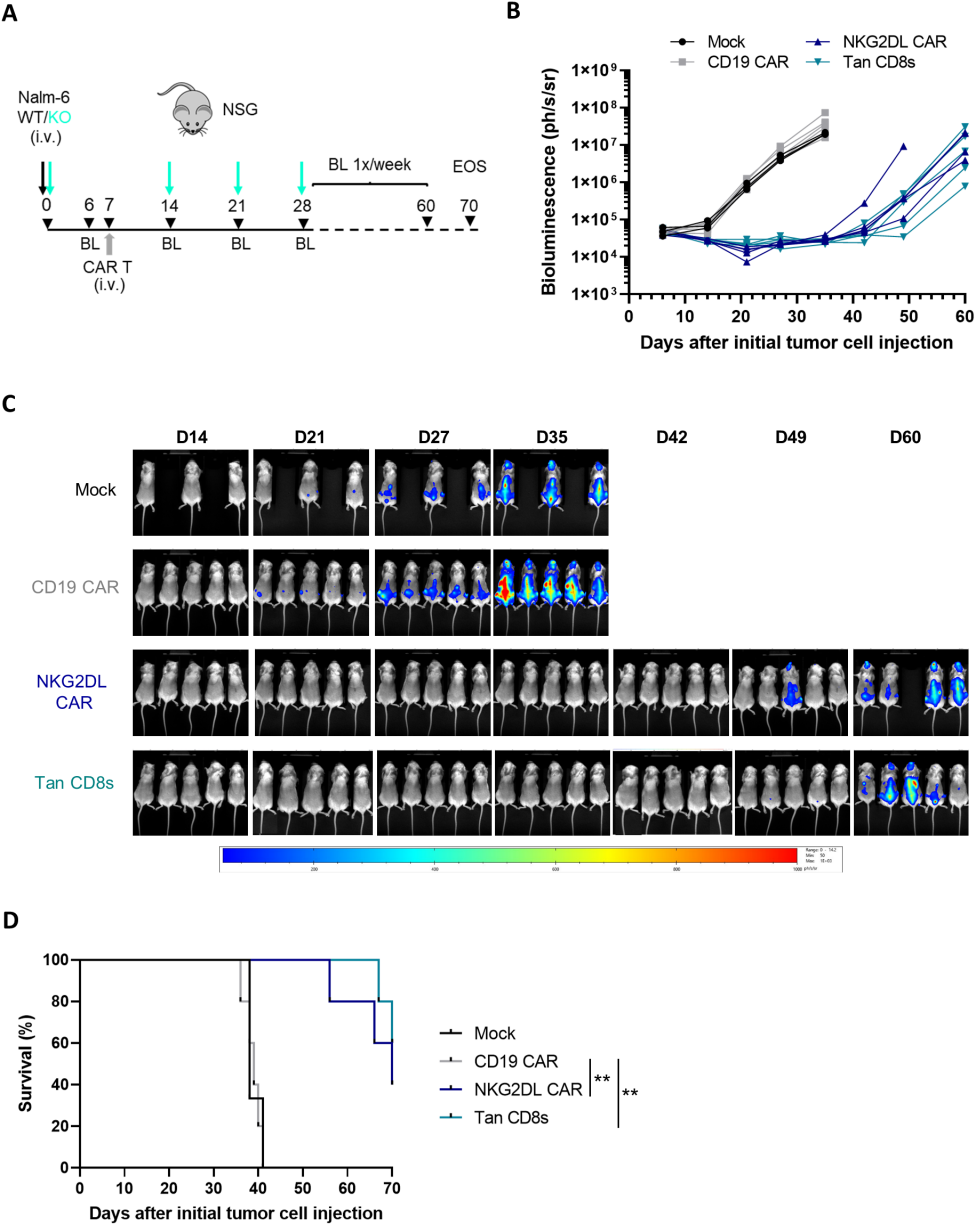
*In vivo* anti-tumor activity of CD19/NKG2DL tandem CD8s CAR T-cells in a B-ALL relapse model. **(A)** The B-ALL relapse model was established in NSG mice by injecting 0.1x10^6^ Nalm-6 tumor cells (mix WT: CD19 KO 1:1) on day 0, followed by an injection of 5x10^6^ CAR T-cells on day 7, and 3 re-challenges of 0.1x10^6^ Nalm-6 CD19 KO cells on day 14, 21 and 28. Study was terminated on day 70. **(B)** Tumor burden in individual mice followed by BLI (n=5, except in mock group n=3) until D60. **(C)** Images depicting the tumor burden monitored by BLI at the indicated time points. **(D)** Kaplan–Meier curves showing survival of NSG mice injected with control, single or tandem CAR T-cells. Adjusted P value (P**<0.005) was determined by Logrank Mantel–Cox test.

We next assessed whether CD19/NKG2DL tandem CAR T-cells with the CD8 short hinge could prevent CD19 antigen escape using an *in vivo* in a B-ALL relapse model. NSG mice were intravenously (i.v.) injected with a 1:1 mix of Nalm-6 cells and Nalm-6 CD19 KO cells on day 0, followed by treatment with mock, single, or tandem CAR T-cells on day 7. Tumor progression was monitored weekly by bioluminescence imaging until day 60 (**Figure 6A**).

To simulate a worst-case scenario resembling aggressive CD19- relapse in B-ALL patients, mice were rechallenged with Nalm-6 CD19 KO cells 1-, 2- and 3-weeks post CAR T-cell treatment. As expected, CD19 single CAR T-cells failed to control tumor growth after the first rechallenge (**Figures 6B, C**) and all mice succumbed to the tumor within 35 days (**Figure 6D**).

In contrast, survival was significantly extended in mice treated with NKG2DL single and tandem CAR T-cells. In these groups, cancer cells remained undetectable in almost all mice until day 42 (35 days after CAR T-cell infusion), after which residual cancer cells could be detected. Notably, no significant difference was observed between CD19/NKG2DL tandem and NKG2DL single CAR T-cell in this model, suggesting that the expression of NKG2DL on the Nalm-6 cells was sufficient to reduce tumor burden significantly.

### CD19/NKG2DL CD8s tandem CAR T-cells target primary B-ALL cells

**Figure 7:**
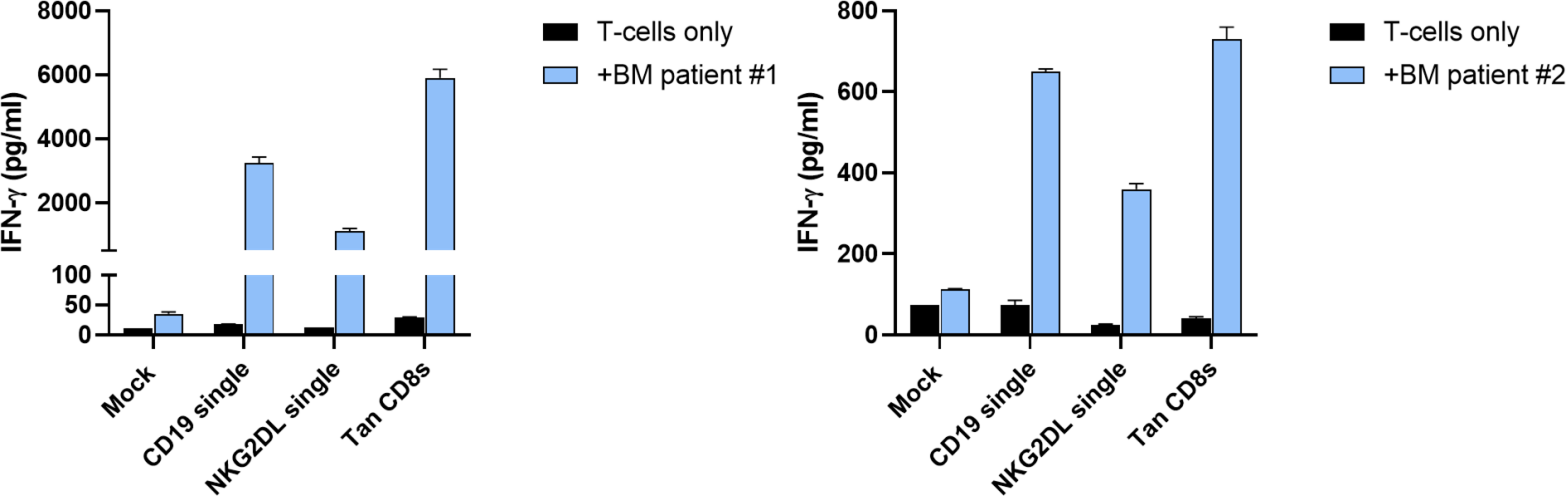
*In vitro* activity of CD19/NKG2DL tandem CD8s CAR T-cells against primary B-ALL bone marrow samples. Secretion of IFN-γ after 24 hours of co-culture at 1:1 E:T ratio with bone marrow specimen from two B-ALL patients. Data presented as means (SD) of n=2 technical replicates.

To determine whether CD19/NKG2DL tandem CAR T-cells can effectively target primary B-ALL tumor cells, we incubated T-cells expressing the tandem with the CD8 short hinge, alongside mock and single CAR T-cells, with CD19+ bone marrow specimen from two B-ALL patients (Immunophenotyping available in **Table 1**). After 24 hours, supernatants were collected for IFN-γ secretion analysis. As shown on **Figure 7**, tandem CAR T-cells secreted IFN-γ at levels comparable to or slightly higher than CD19 CAR T-cells. Additionally, NKG2DL CAR T-cells also produced IFN-γ when co-cultured with primary B-ALL cells, albeit at lower levels. These results validate the potential of NKG2DL targeting in B-cell malignancies, including B-ALL.

**Table 1:** Immunophenotyping of B-ALL bone marrow specimens.

Patient	B-ALL subtype	% tumor cells in BM specimen	B-cell immunophenotyping
#1	unknown	90,7	CD34+, CD45+(dim), CD19+, CD10+, CD22+, CD24+, CD99+, CD33+, HLADR+, Tdti+, cCD79a+
#2	common	94,5	CD34+, CD45-, CD19+, CD10+, CD20+, CD22+, CD38+, HLADR+

## Discussion

In B-cell malignancies, despite high remission rates after CD19 CAR T-cell therapy, a significant percentage of patients eventually relapses due to CD19 loss or downregulation. This is compounded by the existence of CD19 negative clones even prior to treatment with CD19 CAR-T (34, 35). Targeting multiple antigens from the start may reduce the risk of relapse compared to single CAR T-cell therapies. Evaluation of differences in clinical efficacy for single and multispecific CAR T-cells is challenging due to the lack of direct comparison in many studies. Indeed, most multispecific CAR T-cells are only tested in small phase I clinical trials that do not include a CD19 single CAR arm (36–41). Several meta-analyses demonstrated that remission rates of B-ALL and B-NHL patients treated with CD19/CD22 multispecific CAR T-cells were not substantially improved when compared to treatment with single-targeting CAR T-cells (42, 43). Nevertheless, the incidence of antigen-negative relapse observed with monospecific treatments seemed to decrease. As an example, antigen-negative relapses with a CD19^-^/CD22^dim^ phenotype concerned only 6% of relapsing patients (42), against 7-25% (7-9% for adult, and 18-25% for pediatric B-ALL) for CD19 monospecific CAR-T treatments (6, 44). Likewise, antigen-negative relapse in CD19/CD20 CAR T-cells treated B-NHL patients was described for only one patient (38). In other clinical studies assessing CD19/CD22 tandem CAR T-cells, some patients relapsed with cancer cells that were negative for CD19, while CD22 expression was conserved (37, 45) or diminished (46). These results indicate that, while tandem CAR T-cells comprising CD19/CD20 and/or CD19/CD22 are being tested, no clear clinical benefit is apparent, and more data is needed to ensure the validity of the approach.

The rather limited benefit observed with multispecific CAR T-cell therapies targeting two different B-cell antigens might be due to relatively low number of patients treated, the possible presence/emergence of double negative cancer cells, or to the fact that these tandem CARs are not sensitive enough to low antigen densities. The ability of NKG2D to bind eight different tumor-specific ligands with a moderate affinity offers a broad range of targets. In addition, this approach demonstrated a manageable safety profile in > 100 patients evaluated in multiple phase I clinical trials using both autologous and allogeneic NKG2D-based CAR T-cells (47, 48).

In this study, with the goal to prevent CD19 antigen escape in B-cell malignancies, we created different CD19/NKG2DL tandem CAR T-cells and demonstrated that the tandem candidate carrying the CD8 short hinge provided robust and sustained antitumor activity both *in vitro* and *in vivo*, under stress conditions. Specifically, we have shown that these tandem CAR T-cells were able to lyse CD19+ and CD19- cancer cells in both repeated antigen stimulation assays (mimicking chronic stimulation) and in an aggressive *in vivo* B-ALL model where tandem CAR T-cells successfully negated cancer relapse after multiple re-challenges. Finally, we demonstrated that tandem CAR T-cells with the CD8 short hinge, but also single-targeting counterparts, secreted IFN-γ when co-cultured with primary B-ALL samples, highlighting the relevance of targeting NKG2DL in B-cell malignancies, including B-ALL.

Several studies comparing tandem CARs containing two scFv’s to their single-targeting counterparts showed that tandem receptor displayed a lower binding to one or both target antigens, leading to impaired recognition of tumor cells (13–15, 37). In our experiments, CD19 binding was fully conserved while MICA binding was decreased by 30% in CD19/NKG2DL tandem receptors bearing a short hinge as compared to their single targeting counterparts, suggesting that the FMC63 scFv generates some steric hindrance towards the NKG2D extracellular domain. This phenomenon, which was not abrogated by increasing linker size, did not translate in a decreased functionality since T-cells expressing these tandem receptors were as effective as NKG2DL single CAR T-cells in terms of cytokine secretion, proliferation, and cytolytic activity. Moreover, the binding of MICA did not decrease the binding of CD19 and *vice versa*.

In our studies, tandem CAR T-cells with a long hinge, despite similar MICA binding, functioned poorly without CD19 antigen. This highlights the importance of hinge size over NKG2DL binding for functionality. The short extracellular spacer of natural NKG2D suggests that NKG2D-based CAR receptors require a short hinge for effective signaling. Furthermore, while many studies have assessed tandems that differ by the linker size, the linker sequence or the relative position of the scFv’s (14, 49, 50), the impact of hinge size and hinge origin in CD19-targeting tandem receptors has been less commonly addressed (13).

Interestingly, the activity of tandem long hinge CAR T-cells in response to plate-bound MICA did not fully replicate the results observed with Nalm-6 CD19 KO cells. Several factors may contribute to this discrepancy. First, epitope accessibility is likely higher for immobilized ligands compared to the same ligands embedded within a cellular membrane. Second, even at low coating concentrations, the number of MICA molecules available to T-cells may greatly exceed the density of NKG2DL present on the surface of Nalm-6 cells. Third, while the plate-bound system presents a single defined ligand, Nalm-6 cells express multiple NKG2DL simultaneously. Finally, given that the natural receptor NKG2D exhibits varying affinities for its different ligands, the cumulative avidity and resulting activation threshold may differ, depending on the hinge length, potentially leading to distinct responses between T-cells expressing the tandem long hinge and tandem with shorter hinges.

Consistent with this complexity, CAR T-cells with the tandem long hinge and those with shorter hinges displayed comparable cytolytic activity against HeLa cells during the first two stimulations of the repeated killing assay. The presence of high-affinity ligands like MICA on HeLa cells may enhance the activation of long hinge CAR T-cells better than when they are activated by the ULBP ligands expressed at the surface of Nalm-6 cells.

The expression of NKG2DL in primary B-cell malignancies, and particularly in B-ALL, is relatively low in comparison to levels observed in solid tumors. Regulation mechanisms of the stress-associated NKG2DL have been extensively studied and it was demonstrated that their surface expression can be induced by different types of stress conditions such as genotoxic drugs or DNA damaging agents mostly at the post-transcriptional stages (16). Moreover, different cancer treatments such as DNA -damaging agents or histone deacetylase inhibitors were shown to significantly increase their expression at the surface of cancer cells (22, 53). Therefore, the potential of NKG2D-based multispecific CAR-T therapies in B-cell malignancies might be improved by the patient’s pre-conditioning regimen, standard prior treatment lines (e.g. vincristine and doxorubicin) or by combining NKG2DL CAR T-cells with an anti-cancer treatment that increases NKG2DL expression on the surface of cancer cells (e.g. bortezomib and valproate).

By targeting NKG2DL rather than a second B-cell antigen, CD19/NKG2DL tandem CAR T-cells have the potential to eliminate CD19 negative clones arising from lineage switch. These clones, which may be present prior to CD19 CAR-T treatment, carry abnormalities typically associated with myeloid neoplasms, which may then develop into AML (10, 11, 54). This suggests that developing CD19/NKG2DL tandem CAR T-cells may be a good strategy to prevent CD19 antigen-escape resulting from lineage switch as well.

Importantly, in the *in vivo* model used in this study, which aimed to demonstrate the capacity of CD19/NKG2DL tandem CAR T-cells to control CD19- tumor cells, NKG2DL single and tandem CAR T-cells demonstrated comparable anti-tumor efficacy. While this result might suggest that a NKG2DL-targeting CAR could suffice, several key considerations support the continued development of the tandem approach. First, NKG2DL single CAR T-cells secreted lower cytokines levels when co-cultured with Nalm-6 and primary B-ALL samples. Then, CD19-targeting CAR T-cells have a well-established clinical track record and have shown robust efficacy across a range of B-cell malignancies. However, loss or mutation of CD19 often arises under immunological pressure during treatment. The tandem CAR strategy is therefore designed to extend the reach of CAR T-cells by targeting malignant cells that escape CD19-directed therapy. Moreover, the use of an scFv (FMC63) and a natural receptor (NKG2D EC) plays an important role in the design of the CD19/NKG2DL tandem. The FMC63 scFv exhibits a high affinity for CD19, enabling strong and specific binding to CD19 on B-cells. However, its avidity is limited to monovalent interactions, which can impact binding stability, especially in the presence of low antigen density (51). In contrast, natural NKG2D, with its moderate affinity for multiple ligands, benefits from enhanced avidity due to its ability to engage multiple targets simultaneously (52). By leveraging the high specificity of the FMC63 scFv and the broad, multivalent recognition capacity of NKG2D, the tandem CAR is uniquely positioned to enhance targeting across diverse tumor contexts, particularly those exhibiting heterogeneous or downregulated antigen expression.

While the present study focused on B-cell malignancies and used B-ALL as a model to provide the proof-of-concept of NKG2DL multispecific CAR T-cells, the broad expression profile of NKG2DL in cancer implies that it can be combined with different scFv’s to target different indications. In most solid tumors, NKG2DL were shown to be highly expressed [reviewed in (22, 55)]. For example, more than 80% of primary ovarian tumors appear to be positive for at least one NKG2D ligand among MICA, MICB, ULBP1, ULBP2 and ULBP3 (56–58). Colorectal and breast cancer tumor cells were also frequently stained for multiple NKG2DL, implying that these tumor indications would be highly susceptible to NKG2DL mediated lysis (59–63). In addition, we have observed that fibrovascular structures associated with tumors displayed membranous staining within the endothelial compartment suggesting that NKG2D-based CAR-T therapy can target simultaneously both the tumor and the tumor microenvironment (63).

In conclusion, we designed CD19/NKG2DL tandem CAR T-cells that proved to be highly effective against B-cell malignant cells, where low antigen expression or antigen loss can play an important role.

## Data Availability

The raw data supporting the conclusions of this article will be made available by the authors, upon request.
