# Supplementary figures and images for: Tandem CAR T-cells targeting CD19 and NKG2DL can overcome CD19 antigen escape in B-ALL
Source: Front Immunol. 2025 May 9;16:1557405. doi: 10.3389/fimmu.2025.1557405 (PMC12098294; doi:10.3389/fimmu.2025.1557405)

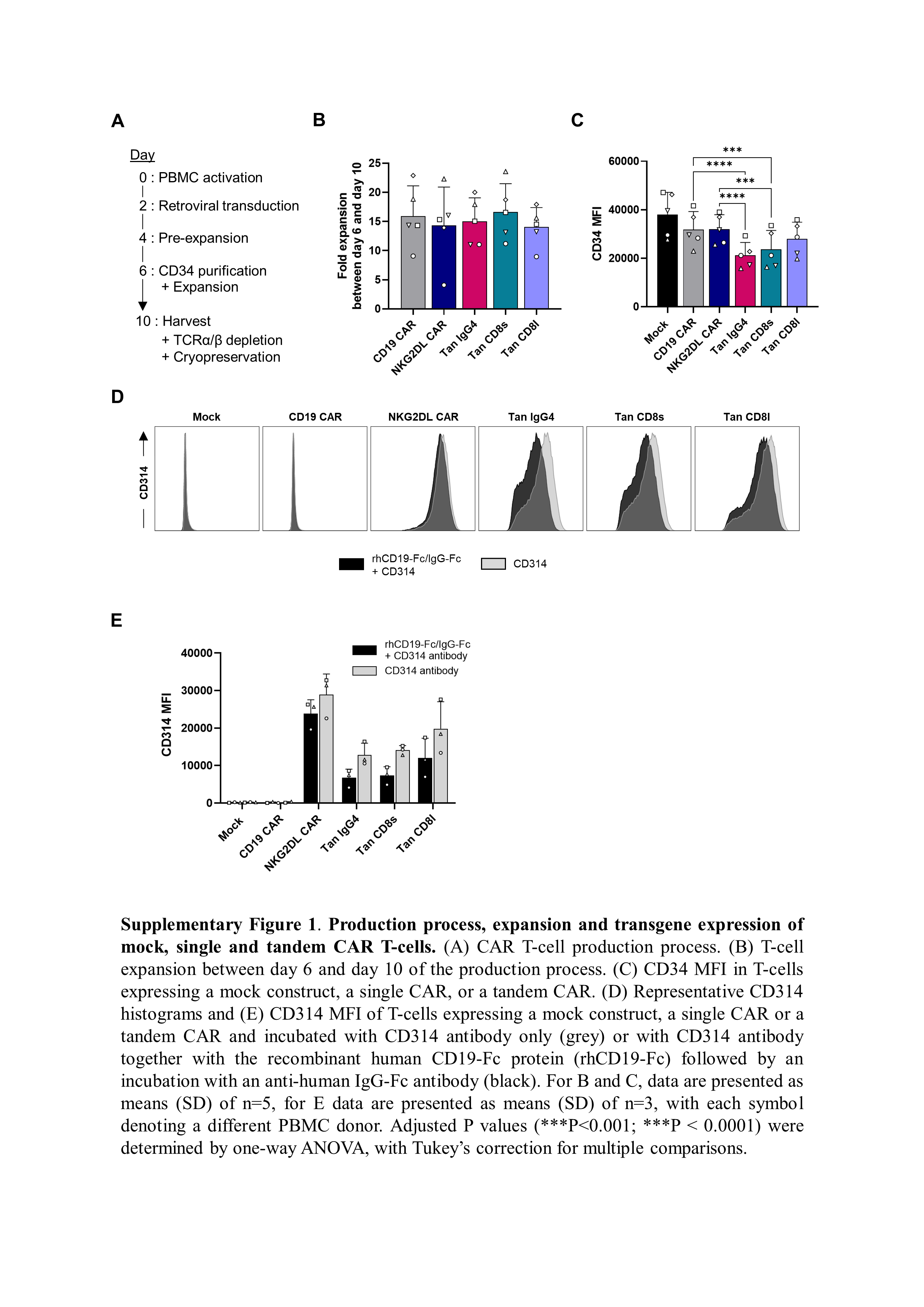

Supplement: Supplementary file 1 [file Image1.tif]

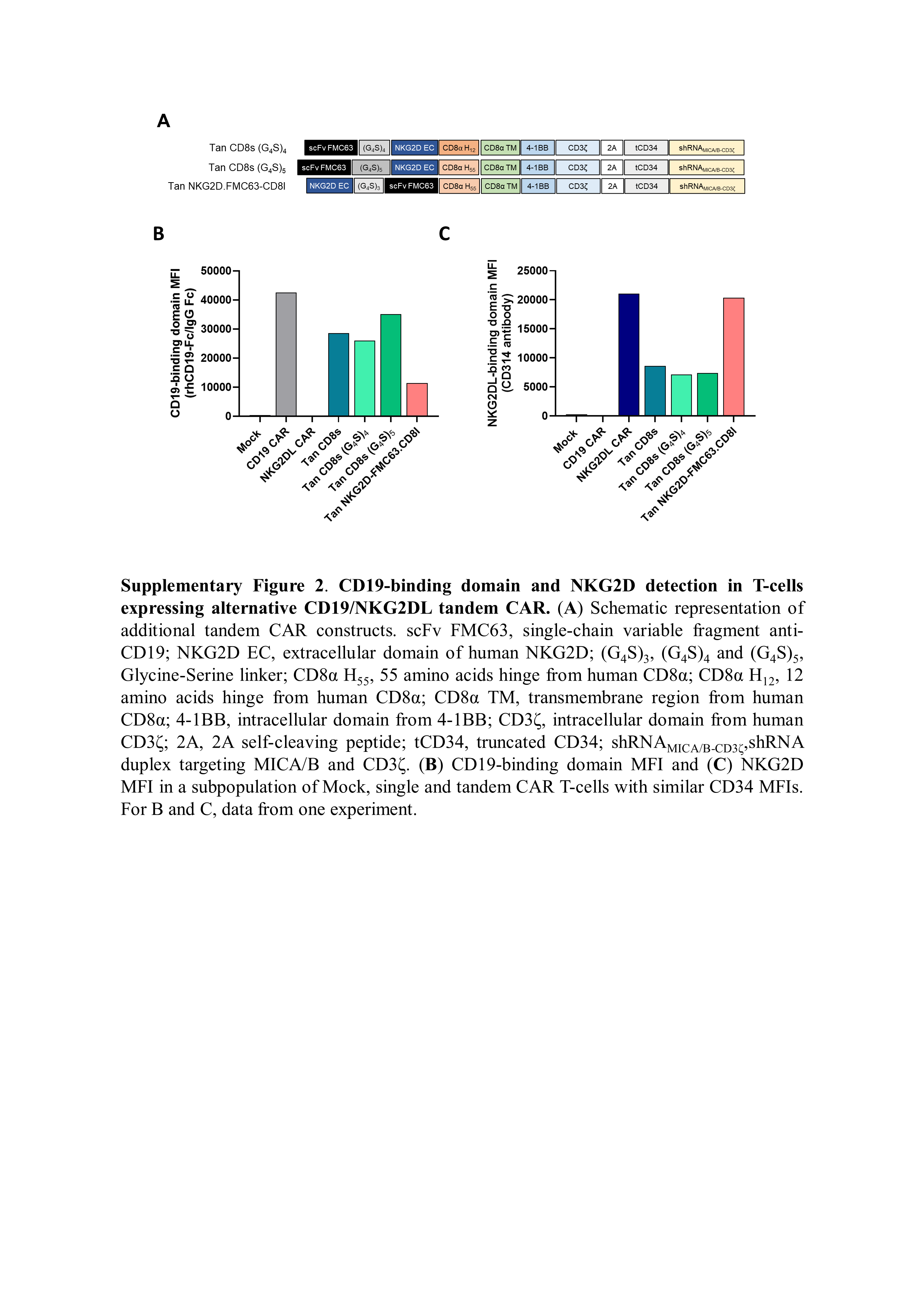

Supplement: Supplementary file 2 [file Image2.tif]

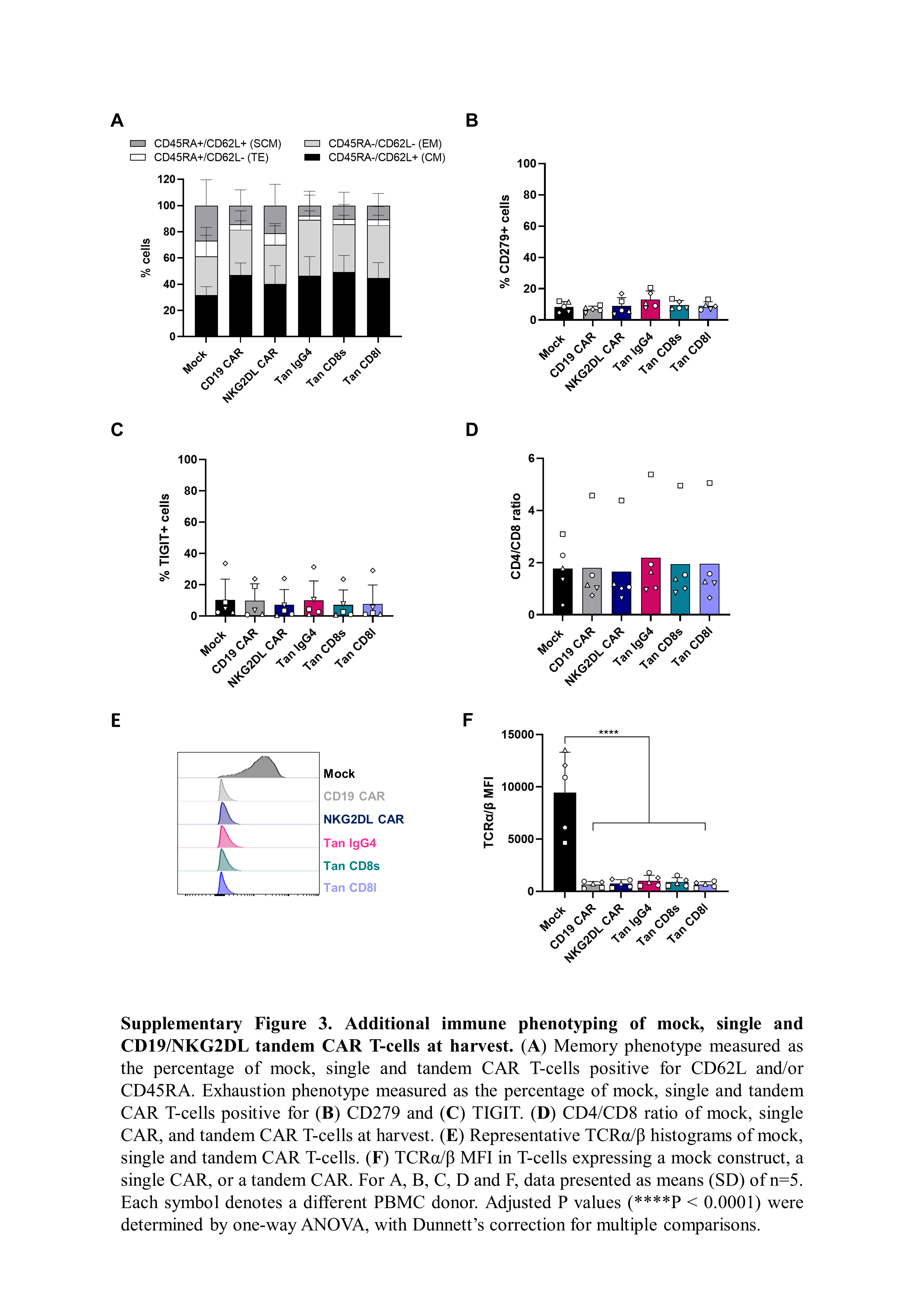

Supplement: Supplementary file 3 [file Image3.tif]

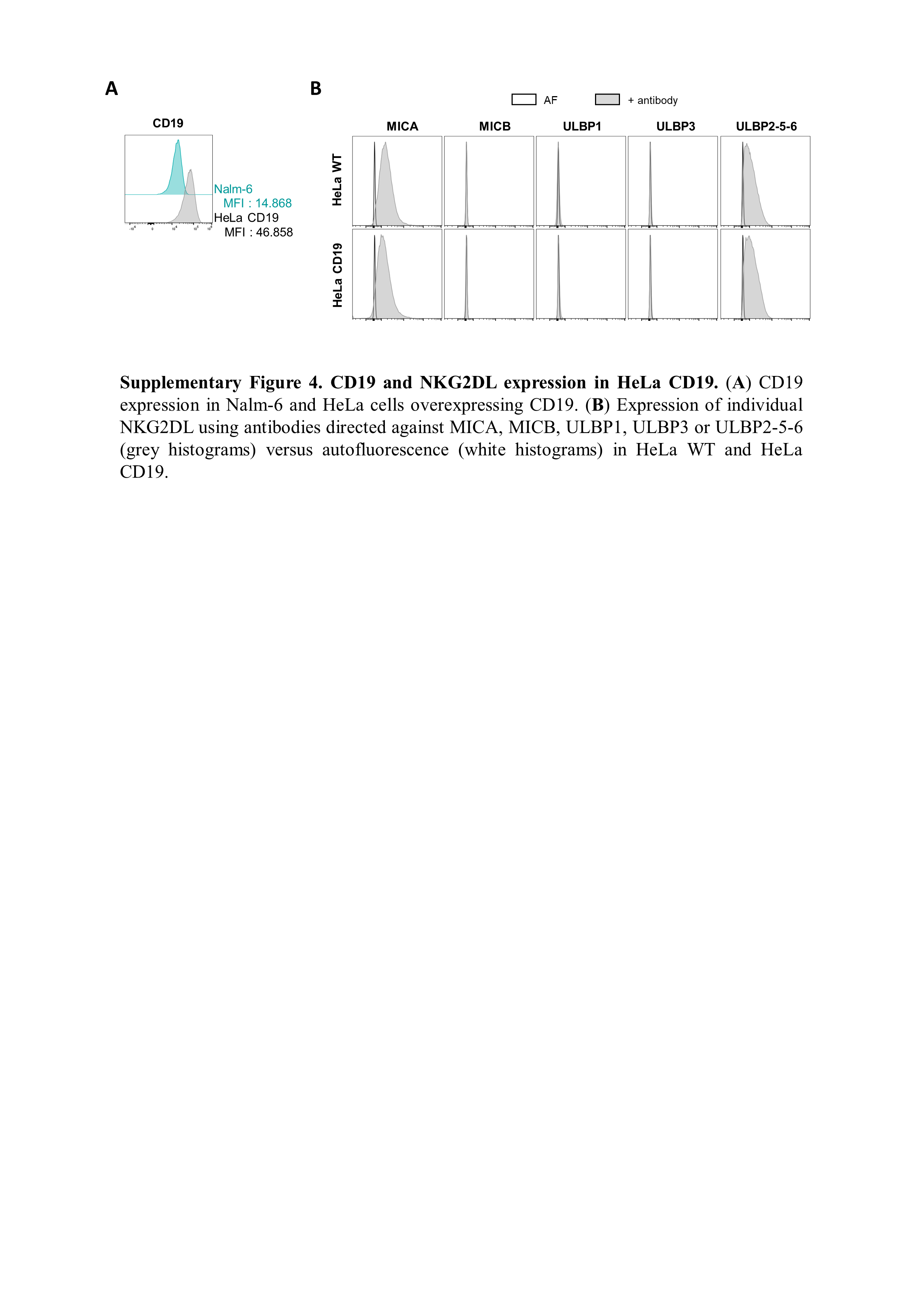

Supplement: Supplementary file 4 [file Image4.tif]
